# Characterization of a 2.5 MV inline portal imaging beam

**DOI:** 10.1120/jacmp.v17i5.6323

**Published:** 2016-09-08

**Authors:** James L. Gräfe, Jennifer Owen, J. Eduardo Villarreal‐Barajas, Rao F.H. Khan

**Affiliations:** ^1^ Department of Physics Faculty of Science, Ryerson University Toronto ON Canada; ^2^ Department of Medical Physics Tom Baker Cancer Centre Calgary AB Canada; ^3^ Department of Physics and Astronomy University of Calgary Calgary AB Canada; ^4^ Department of Oncology University of Calgary Calgary AB Canada; ^5^ Department of Radiation Oncology Medical Physics Division, Washington University School of Medicine St. Louis MO USA

**Keywords:** portal imaging, 2.5 MV, dosimetry, image quality, radiation beam quality

## Abstract

A new megavoltage (MV) energy was recently introduced on Varian TrueBeam linear accelerators for imaging applications. This work describes the experimental characterization of a 2.5 MV inline portal imaging beam for commissioning, routine clinical use, and quality assurance purposes. The beam quality of the 2.5 MV beam was determined by measuring a percent depth dose, PDD, in water phantom for $10\times 10\;{\text{cm}}^{2}$ field at source‐to‐surface distance 100 cm with a CC13 ion chamber, plane parallel Markus chamber, and GafChromic EBT3 film. Absolute dosimetric output calibration of the beam was performed using a traceable calibrated ionization chamber, following the AAPM Task Group 51 procedure. EBT3 film measurements were also performed to measure entrance dose. The output stability of the imaging beam was monitored for five months. Coincidence of 2.5 MV imaging beam with 6 MV therapy beam was verified with hidden‐target cubic phantom. Image quality was studied using the Leeds and QC3 phantom. The depth of maximum dose, $d_{\max}$, and percent dose at 10 cm depth were, respectively, 5.7 mm and 51.7% for CC13, 6.1 mm and 51.9% for Markus chamber, and 5.1 mm and 51.9% for EBT3 film. The 2.5 MV beam quality is slightly inferior to that of a ${\;}^{60}\text{Co}$ teletherapy beam; however, an estimated $k_{Q}$ of 1.00 was used for output calibration purposes. The beam output was found to be stable to within 1% over a five‐month period. The relative entrance dose as measured with EBT3 films was 63%, compared to 23% for a clinical 6 MV beam for a $10\times 10\;{\text{cm}}^{2}$ field. Overall coincidence of the 2.5 MV imaging beam with the 6 MV clinical therapy beam was within 0.2 mm. Image quality results for two commonly used imaging phantoms were superior for the 2.5 MV beam when compared to the conventional 6 MV beam. The results from measurements on two TrueBeam accelerators show that 2.5 MV imaging beam is slightly softer than a therapeutic ${\;}^{60}\text{Co}$ beam, it provides superior image quality than a 6 MV therapy beam, and has excellent output stability. These 2.5 MV beam characterization results can serve as reference for clinics planning to commission and use this novel energy‐image modality.

PACS number(s): 87.57.‐s, 87.59.‐e, 06.20.fb, 87.53.Bn

## I. INTRODUCTION

Recently, low‐energy megavoltage imaging with new target designs attained a renewed interest. Decreasing the nominal incident electron beam energy and atomic number of the target material effectively softens the photon spectrum, which increases contrast and resolution of MV radiographs. A new low‐energy 2.5 megavoltage (MV) imaging beam was made available for in‐line, portal‐image verification of patient setup on the TrueBeam linear accelerator (Varian Medical Systems, Palo Alto, CA). This beam possesses a lower average photon energy and in theory should produce better soft‐tissue contrast due to a larger proportion of diagnostic‐quality photons in the beam spectrum compared to therapy beams (e.g., 6 MV). The 2.5 MV beam is produced by a lower energy (2.5 MeV) electron beam focused on a 2 mm Cu target*. Coincidence of the therapy and kV imaging isocenters and the radiation treatment isocenter is essential for high‐precision, image‐guided radiotherapy. The locations and sizes of these isocenters can differ for various reasons such as gantry sag, uncertainty in the calibration of the imaging arms, and mechanical sag in the imaging arms. A highly accurate and efficient quality assurance (QA) is required to verify the coincidence of the MV and kV imager centers with the treatment isocenter. By design, the 2.5 MV beam is in line with the treatment beams having the potential to remove some of the isocentre coincidence uncertainties that can occur with orthogonal kV imagers mounted on linear accelerators. However, for this to be true, the 2.5 MV beam must be perfectly aligned with the treatment isocenter. Unlike other traditional megavoltage radiotherapy beams, the 2.5 MV beam operates without a flattening filter at a single nominal dose rate of 60 Monitor Units (MU) per minute. Some details of development of this type of beam can be found in references.^1^, ^2^

The 2.5 MV dedicated imaging beam is unlike other image‐guided radiation therapy (IGRT) devices such as the orthogonal kV imagers, as this beam is controlled by the monitor chambers of the linac. Therefore this beam must be calibrated in the same way as the treatment beams. There are currently no guidance documents for calibrating this beam with a lower quality than the conventional ${\;}^{60}\text{Co}$ teletherapy beam used in standards labs and following the TG‐51 procedure.

There are several Monte Carlo simulation studies involving low atomic number (Z) targets in Varian,^1^, ^2^, ^3^ Elekta,^4^, ^5^ and Siemens^6^, ^7^ linacs. However, a literature review on this subject reveals lack of experimental data and guidance leading to clinical use of this important new modality. In particular, there is no documentation regarding practical quality assurance testing applicable to this novel imaging modality. In this work, we have addressed some of these issues by characterizing the imaging beam from a dosimetry and beam quality perspective. This characterization includes a set of beam data from the commissioning and quality assurance test results that will facilitate the clinical implementation of this novel MV‐imaging beam.

The challenges in terms of absolute beam output calibration, determination of dosimetric parameters, and verification of coincidence of the 2.5 MV beam with the 6 MV treatment beam are described in following sections. We also present some preliminary image quality comparison results using commercial imaging phantoms, namely the Leeds TOR 18FG phantom (Leeds Test Objects Ltd., UK) and the QC3 phantom (Standard Imaging Inc., Middleton, WI).

## II. MATERIALS AND METHODS

All measurements were performed on two recently commissioned Varian TrueBeam linear accelerators equipped with a 2.5 MV photon imaging beam (hereafter referred to as TB1 and TB2) in our clinic. Only TB1 is equipped with a new $43\times 43\;{\text{cm}}^{2}$ digital megavolt imager (DMI); all image quality tests were performed on this detector.

### A. Beam characterization: ion chamber measurements

#### A.1 Percentage depth‐dose measurements and beam profiles

The percentage depth‐dose (PDD) curves were measured with both a $0.13\;{\text{cm}}^{3}$ CC13 (Scanditronix Wellhöfer, Nuremburg, Germany) and a $0.055\;{\text{cm}}^{3}$ Markus plane‐parallel ion

chamber, model no. 23343 (PTW Frieburg, Germany) in a water tank (Wellhöfer Scanditronix Blue Phantom) set up at source‐to‐surface distance (SSD) of 100 cm with a $10\times 10\;{\text{cm}}^{2}$ field. The PDD curve was shifted to take into account the effective point of measurement of the two chambers (1.8 mm for CC13, 1 mm for Markus chamber). The PDD was also measured with radiochromic film, as described in section B.1. The tissue maximum ratio (TMR) was also measured for $10\times 10\;{\text{cm}}^{2}$ field at depth of 10 cm in water phantom.

The beam profiles were measured with a CC13 ion chamber at 100 SSD in the water phantom for a maximum field size of $40\times 40\;{\text{cm}}^{2}$ at 5 cm depth in water for 2.5 MV, 6 MV, and 10 MV‐FFF beams. The measurements were performed in both in‐plane and cross‐plane directions.

#### A.2 Reference dosimetry

The reference dose was measured using a $0.64\;{\text{cm}}^{3}$ Exradin A12 thimble ionization chamber (Standard Imaging Inc., Middleton, WI) and a Standard Imaging SuperMAX electrometer, with calibration traceable to the National Research Council of Canada (NRCC). The absolute dosimetry was performed according to the AAPM TG‐51 calibration procedure^(8)^ using a source‐to‐axis distance (SAD) setup under reference conditions. The TG‐51 protocol specifies the quality conversion factor, $k_{Q}$ for beams with quality determined by $\%\text{dd}{\left(10\right)}^{x}$ as a means of converting the chamber response for the user's beam to that of the reference beam quality. The reference beam quality in standards laboratories is that of ${\;}^{60}\text{Co}$. The beam quality for ${\;}^{60}\text{Co}$ is a $\%\text{dd}{\left(10\right)}^{x}$ of 58%. The TG‐51 protocol specifies the $k_{Q}$ values for beam qualities higher than ${\;}^{60}\text{Co}$. However, the measured $\%\text{dd}{\left(10\right)}^{x}$ of approximately 52% for the 2.5 MV beam is slightly lower quality. For calibration purposes, we chose to use a $k_{Q}$ of unity (i.e., that of ${\;}^{60}\text{Co}$). This value was determined to be a reasonable approximation as the ratios of the restricted stopping power ${\left(\frac{L}{\rho }\right)}_{\text{air}}^{\text{water}}$, for 2 MV and ${\;}^{60}\text{Co}$ are approximately the same to within 0.4%.^(9)^

The 2.5 MV outputs of the two TrueBeam accelerators were calibrated so that 1 MU delivered 1 cGy at $d_{\max}$ with a $10\times 10\;{\text{cm}}^{2}$ field at 100 SAD. To achieve this output, the AAPM TG‐51 procedure was performed under SAD conditions with the chamber placed at a depth of 10 cm. The measured TMR (10×10, d=10) was applied to determine the dose at $d_{\max}$ and the beam was adjusted to achieve 1 cGy/MU at $d_{\max}$. The outputs were measured on the same day using a Capintec PR‐06C ion chamber (Capintec Inc., Ramsey, NJ) at a depth 10 cm in a Solid Water (Gammex RMI, Middleton, WI) with 6 cm used for backscatter. These output values were then measured weekly for the first month and monthly afterwards, in order to monitor the 2.5 MV beam output consistency.

### B. Radiochromic film measurements

EBT3 radiochromic film (Ashland, Bridgewater, NJ) was used for all film measurements. Films were scanned in 48‐bit red‐green‐blue (RGB) format using an EPSON Expression 10000 XL flatbed scanner (Epson America Inc., Long Beach, CA) at 72 dpi in transmission mode with no color or sharpness correction. The film orientation was marked and templates were used to reproducibly position the film on the scanner. Each film was scanned four times, with the fourth scan used for results. This was done to allow the scanner to warm up and stabilize. Details of our scanning and calibration procedure have been described previously.^(10)^ The net optical density (netOD) was determined from the red channel information.

A calibration curve with netOD vs. absorbed dose was produced using films that were given known doses with a therapeutic 6 MV beam previously calibrated following the AAPM TG‐51 protocol. We assumed no energy‐dependent response of the EBT3 film for the 2.5 MV beam compared to the 6 MV beam.

#### B.1 Percentage depth‐dose measurements

An approximately $17.5\times 10\;{\text{cm}}^{2}$ strip of EBT3 film was suspended vertically (parallel to the beam axis) in a small water tank using a custom‐built acrylic film holder. The film edge was aligned with the surface of the water centered in a $10\times 10\;{\text{cm}}^{2}$ field at 100 SSD. The film was irradiated with the 2.5 MV beam from the TrueBeam accelerator.

A 30‐pixels‐wide (corresponding to a 1 cm region, uniform dose to within 0.5%) strip running vertically down the center of the film was used for the dose measurement. For each horizontal row of 30 pixels, an average transmission value was used to obtain the netOD and converted to dose using the calibration curve. The PDD was then determined by normalizing the dose to the measured $d_{\max}$.

#### B.2 Entrance dose measurements

The entrance dose for 2.5 MV beam $10\times 10\;{\text{cm}}^{2}$ field was measured using a $4\times 4\;{\text{cm}}^{2}$ film at 100 SSD placed directly on top of 10 cm of Solid Water for backscatter, with no buildup material above. The effective depth of measurement for the EBT3 film lying flat on a surface orthogonal to the beam is in the middle of the active layer, about 120 μm. With a density of approximately $1.35\;g/{\text{cm}}^{3}$ this corresponds to a radiological effective depth of approximately 162 μm. The film measurements were repeated for the 6 MV radiotherapy beam used for the EBT3 film calibration. Based on experience with EBT3 film dosimetry in our clinic, we assume a 5% dosimetric uncertainty on all EBT3 measurements, due to error propagation from calibration, film handling, and scanning.

### C. Coincidence of imaging and therapy beams

For a rigorous assessment of the beam center coincidence between 2.5 MV imaging and 6 MV therapy beam, ImageProWL‐QA phantom (Sun Nuclear Corporation, Melbourne, FL), a cube which contains a radiopaque sphere in the center was used. This test is based on the procedure developed by Nathan Childress et al. (distributed via http://www.medphysfiles.com/). The phantom was first imaged on a Philips BigBore CT scanner with 1 mm slice thickness (Philips Healthcare, Andover, MA), and the images imported into the Eclipse treatment planning software (Varian). On the treatment unit, the phantom was set up at isocenter with the help of in‐room lasers and verified using a 2D/2D MV‐MV match image registration with the 2.5 MV imaging beam, performing any shifts needed. Images were then taken with both the 2.5 MV and 6 MV beams at multiple gantry, collimator, and couch angles (Table 1). For each orientation, the 2.5 MV image was acquired, followed by the 6 MV image, so that reproducibility of the machine position should not bias the results.

**Table 1:** Gantry, collimator, and table rotations used in Winston‐Lutz test for imaging and therapy isocenter verification.

*Gantry Angle (°)*	*Collimator Angle (°)*	*Table Angle (°)*
0	90	0
0	270	0
90	90	0
90	270	0
180	270	0
270	270	0
0	270	30
0	270	90
0	270	60
0	270	270
0	270	300
0	270	330

The images were imported into DoseLab Pro (Mobius Medical Systems, Bellaire, TX) and analyzed by the Winston‐Lutz module. The algorithm compares the position of the centroid of the radiation field with that of the radiopaque sphere, which is presumed to be centered on isocenter. The displacement from the radiation center to the sphere is calculated for both horizontal and vertical directions in the image. As these images were obtained with both the 2.5 and 6 MV beams, the displacements calculated for each energy at each orientation were compared to each other. A correlation in displacements between the two energies would indicate the systematic difference in location of the isocenter for both the treatment and the imaging.

### D. Image registration

The 2.5 MV imaging beam operates in conjunction with electronic portal imager on a TrueBeam treatment unit. The primary concern regarding the geometry of a portal imaging system is that it can be used to reproduce the planned setup of a patient from CT simulation to within a given tolerance. For this reason, a test of the image registration capabilities was performed with both the 2.5 MV imaging beam and the kV on‐board imaging system for comparison using the Varian cubic (VC) phantom. The VC phantom provided by Varian is a plastic cube and it was modified by adding tungsten wires to the surface. The verification method is a common QA procedure used to compare the MV and kV isocenters with the mechanical isocenter in clinical practice. The VC phantom was first simulated on a Philips BigBore CT scanner and the images imported into the Eclipse treatment planning software. The VC phantom was aligned to the machine's mechanical isocenter using room lasers and/or the projection of the machine's light‐field crosshairs. The linac couch was then shifted so that the phantom was displaced from the isocenter by a known distance of 0, or ±2 cm in either the longitudinal, lateral, or vertical direction. The couch motion accuracy was verified prior to these measurements to be within ±1 mm.

At this displaced position, orthogonal images of the phantom were first obtained using the 2.5 MV beam and subsequently with the orthogonal kV imaging beam. Using the automatch software treatment couch shifts were determined for the VC phantom to return the isocube to the planned isocenter. Therefore, this allowed us to compare the known physical phantom position to that calculated using the imaging software for the 2.5 MV imaging beam and the kV imaging beam independently.

### E. Image quality

In order to assess the image quality performance of the 2.5 MV beam, we imaged the Leeds TOR 18FG phantom (Leeds Test Objects Ltd., Leeds, UK) and the QC3 phantom. The Leeds phantom was imaged in a similar way as by Song et al.^(11)^ The TOR 18FG phantom was positioned on the couch and thicknesses of Solid Water from 0–20 cm were placed over the phantom. Images were obtained with the 2.5 MV beam (High Quality mode), 6 MV beam (High Quality mode), and kV source at 80 kVp and 0.8 mAs.

For the QC3 phantom we took images in both High Quality and Low Dose mode using the 6 MV and 2.5 MV beams. The images were then analyzed using the Imaging QA module of DoseLab Pro, modulation was plotted as a function of object resolution.

## III. RESULTS AND DISCUSSION

### A. Beam characteristics and absolute calibration

**Figure 1:**
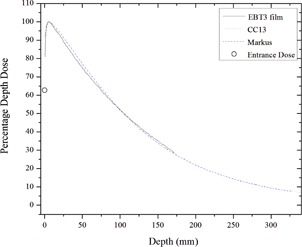
2.5 MV percent depth‐dose in water phantom measured with EBT3 film, CC13 ionization chamber, and Markus plane‐parallel chamber.

The EBT3 measured relative entrance dose was found to be 63% for the 2.5 MV beam for a $10\times 10\;{\text{cm}}^{2}$ field size at 100 SSD. In comparison, the surface dose for the conventional 6 MV portal imaging beam was 23%. When taking into account the effective measurement depth, this latter value compares well with the literature values ranging from 16.4%–20% for Varian TrueBeam machines for a $10\times 10\;{\text{cm}}^{2}$ field size.^12^, ^13^, ^14^ The 2.5 MV entrance dose is somewhat lower than the Monte Carlo simulated value of 81% reported by Parsons et al.^(1)^ A comparison of the PDD measured with EBT3 film, and the CC13 and Markus chambers are shown in Fig. 1. The depth of maximum dose, $d_{\max}$, and percent dose at 10 cm depth were, respectively, 5.7 mm and 51.7% for CC13, 6.1 mm and 51.9% for Markus chamber, and 5.1 mm and 51.9% for EBT3 film. The 2.5 MV imaging beam operates in both High‐Quality (1.5 MU) and Low‐Dose (1 MU) modes. The beam is controlled by the linac MU ionization chamber and demonstrates the potential for optimization of both the dose/MU and image quality of this novel beam.

The 2.5 MV beam has a %dd(10) of ∼52% compared to 53.9% computed by Parsons et al.^(1)^ The TMR (10×10) at 10 cm depth is approximately 0.603. The 2.5 MV exhibited nearly three times higher surface dose than the 6 MV beam (63% compared to 23%); however, for a separation of 20 cm, the 2.5 MV mid‐separation dose is about 78% of the 6 MV dose ($\%\text{dd}{\left(10\right)}_{2.5\text{MV}}=52\%$ and $\%\text{dd}{\left(10\right)}_{6\text{MV}}=67\%$) and the exit dose is only ∼58% of the 6 MV $\text{dose}\;((\%\text{dd}{\left(20\right)}_{2.5\text{MV}}=22\%$ and $\%\text{dd}{\left(20\right)}_{6\text{MV}}=38\%$). Table 2 lists various parameters needed for the absolute calibration under reference condition. A $k_{Q}$ of unity was used, which is in line with IAEA TRS‐398 protocol. In TRS‐398 protocol there is only 0.5% variation of $k_{Q}$ between ${\;}^{137}\text{Cs}$ and ${\;}^{60}\text{Co}$ beam qualities,^15^, ^16^ which further supports our choice of $k_{Q}=1.00$. The calibration procedure described here using TG‐51 is therefore reliable to an uncertainty of approximately 0.5%.

**Figure 2:**
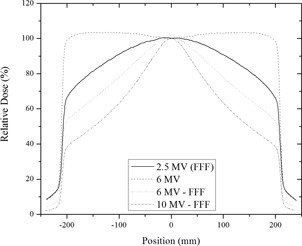
A comparison of crossline beam profiles for the 2.5 MV, 6 MV, 6MV‐FFF, and 10 MV‐FFF beams at depth of > 5 cm in water.

A comparison of the beam profiles for a flattened 6 MV beam and unflattened (FFF: flattening filter–free) 2.5 MV, 6 MV, and 10 MV beams is shown in Fig. 2. The forward‐directed energy dependence of the bremsstrahlung X‐ray production is clearly demonstrated when comparing the 2.5 MV, 6 MV, and 10 MV unflattened beams. The unflatness of the 2.5 MV imaging beam is not as pronounced as the 10 MV unflattened treatment beam.

**Table 2:** Absolute dosimetry, TG‐51 parameters for 2.5 MV beam calibration.

*Parameter*	*2.5MV*
$P_{\text{ion}}$	1.004
$P_{\text{pol}}$	1.000
$d_{\max}$	5.9 mm
%dd(10)	52
$k_{Q}$	1.000
TMR (10×10, depth 10 cm)	0.603

### B. Beam coincidence

**Figure 3:**
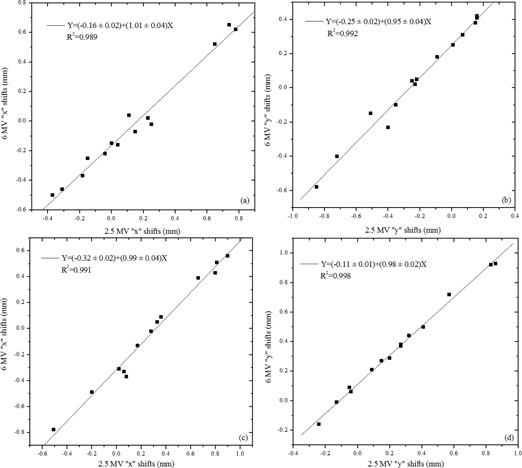
Correlation of the calculated position (“shift”) of the W‐L phantom radiopaque sphere for the 2.5 MV and 6 MV beams on both TrueBeam units (TB1 (a), (b), and TB2 (c), (d)).

The Winston‐Lutz (W‐L) test demonstrates that the 6 MV and 2.5 MV “shifts” or disagreements from the radiation and mechanical isocenters for both beams are well correlated, as shown in Fig. 3. The data plotted in this figure are the calculated discrepancies between the center of the radiation field and the center of the radiopaque ball from the EPID images obtained at various combinations of gantry, collimator, and the table angles. These discrepancies are plotted for the 6 MV against the 2.5 MV beam in order to determine if the deviations from the ideal isocenter are correlated between the two beams. The “x” and “y” shifts in the figure correspond to the shifts in the images recorded by the portal imager. In order to compare the overall isocenter coincidence the software reports the total offsets in three‐dimensional space based on the gantry, collimator, and couch angle obtained by the software from the DICOM image file. These results are shown in Table 3. The total vector shifts from the isocenter coincidence of the 6 MV and 2.5 MV beams are within 0.2 mm for both treatment units. In other words the W‐L tests demonstrates the strong spatial correlation of the two beams and confirms that shifts determined by the 2.5 MV can be applied reliably for treatments using the 6 MV beam.

**Table 3:** 3D displacement of isocenter vector obtained from the W‐L phantom analysis for the 2.5 and 6 MV beams.

*Energy*	*X (mm)*	*Y (mm)*	*Z (mm)*	*Net Vector Displacement (mm)*
*TB1*				
2.5 MV	0.14	−0.4	0.13	0.44
6 MV	0.09	−0.19	0.18	0.28
*TB2*				
2.5 MV	0.09	0.39	0.50	0.64
6 MV	−0.01	0.48	0.57	0.75

### C. Image registration

**Figure 4:**
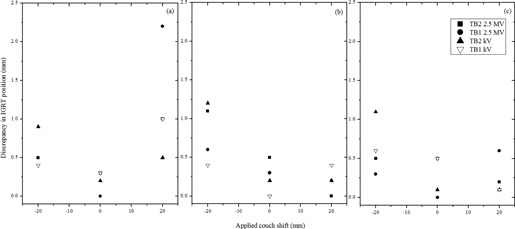
A plot of the discrepancies between the known table shifts of 0, and ±2 cm, for the 2.5 MV imaging beam and kV imager for vertical (a), lateral (b), and longitudinal (c) displacements of the VC phantom from isocenter.

A known shift of 0 or ±2 cm from isocenter was applied to the VC phantom. In Fig. 4, the results of the difference between the applied shift and the auto match calculated shift against the applied shifts are shown. This represents the IGRT software discrepancy in positioning for the 2.5 MV and kV imaging beams. The majority of the software‐calculated shifts were within 1.1 mm for the 2.5 MV imaging beam. The average difference between the known shift for both treatment units was 0.5±0.5 mm. This submillimeter difference is within the current operational standard for the IGRT kV imager.

### D. Image quality

#### D.1 Leeds phantom

**Figure 5:**
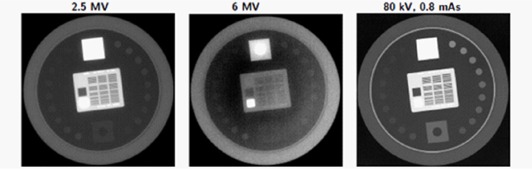
High‐ and low‐contrast objects from Leeds test object phantom without overlaying scatterer.

**Figure 6:**
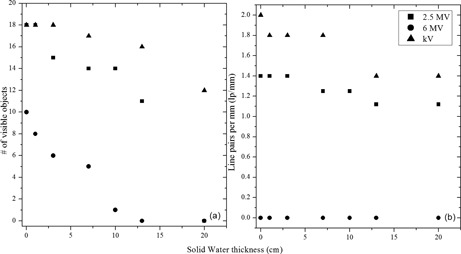
Low‐contrast (a) and high‐contrast (b) resolution as a function of overlying Solid Water thickness for the imaging beams.

Measurements were performed using the TB1 equipped with the DMI detector. The results shown in Fig. 5 demonstrate the superior image quality of the 2.5 MV compared to the 6 MV beam. With no overlaying Solid Water, all of 18 low‐contrast objects are visible on the 2.5 MV beam and the 80 kV OBI beam. Only 10 of 18 of the low‐contrast objects are visible with the 6 MV beam. For the high‐contrast portion of the phantom, the resolution is 1.41p/mm at 2.5 MV and 21p/mm at 80 kV, while none of the pairs are discernible for the 6 MV image. These image quality results are comparable to those observed by Song et al.^(11)^ A plot of the low‐contrast and high‐contrast resolution for the Leeds phantom is shown in Fig. 6. It can be seen that for this phantom the 2.5 MV beam is superior to the 6 MV beam; however, 2.5 MV image quality is at best the same or inferior over various thickness of overlying scatterers when compared to the kilovoltage images.

#### D.2 QC3 phantom

**Figure 7:**
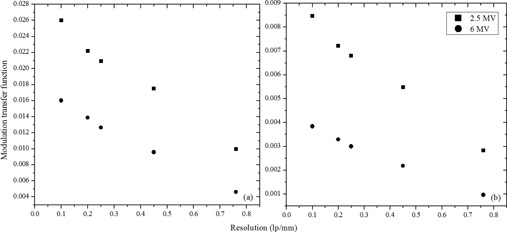
Estimated modulation transfer function for 2.5 MV and 6 MV beams for (a) high‐quality and (b) low‐dose images.

The image quality was assessed with the Standard Imaging QC3 phantom. The estimated modulation transfer function (MTF) for the portal imaging system shows that 2.5 MV has better spatial resolution than a 6 MV beam (Fig. 7). Indeed, this is also evident qualitatively from the Leeds phantom images in Fig. 5.

### E. Quality assurance recommendations

In this work we characterize the novel 2.5 MV imaging beam on Varian TrueBeam linear accelerator both in terms of dosimetric quality and preliminary imaging quality. The imaging beam was found to be stable to within 1% over a period of 5 months. Since beam output and profile are important both in terms of dose and safety, we recommend monitoring them as part of monthly QC activity to within 2% and 2% or 2 mm respectively. Imaging quality in terms of low‐ and high‐contrast objects can be evaluated on a monthly basis with comparisons done relative to baseline or commissioning data. The beam quality, albeit energy in terms of PDD, should be measured annually to within 2% of the reference PDD. In addition, it is recommended that the W‐L isocenter coincidence test be performed annually. However, for machines using this beam for hypofractionated treatment setup, this test should be performed on a routine basis depending on state regulation and local practices in the radiation oncology department.

In this work we characterized a novel, hence unreported, megavoltage imaging beam on Varian TrueBeam linear accelerator and provided baseline data and shared our experience with the medical physics community. Superior image quality was observed for the 2.5 MV beam over the 6 MV beam using the Leeds phantom. However, we could not find a clear trend for contrast and contrast‐to‐noise‐ratio for the QC3 phantom. Therefore we chose not to present those results, but this is an area worth further exploration. We believe that the QC3 phantom MTF results demonstrate clearly the resolution differences between 2.5 MV and 6 MV. The resolution is also demonstrated by the Leeds phantom results, along with the contrast comparisons. The 2.5 MV imaging beam still has to find its clinical utility and relevance in presence of high‐quality kV imaging already in routine clinical use. However, the 2.5 MV beam could be ideal for fast 2D/2D MV/kV matching of patients without rotating the gantry. Megavoltage imaging has an advantage in terms of avoiding streaking artifacts when it comes to imaging high‐density objects such as metal prostheses close to the target volume. The new imaging option could be a useful backup in setting up patients when the kV imaging system is unavailable. In most of the clinics, availability of an MV computed tomography would benefit resolving imaging artifacts in delineating target volume in proximity to high‐Z implants when used in combination with conventional CT simulation. Therefore we foresee further development of an MV CBCT on Varian TrueBeam linear accelerators in future. Unfortunately, at this time CBCT with 2.5 MV mode is not available to us in Canada. This is an option we believe Varian is still working on developing and we would be excited to test in the future. We can speculate on other potential applications in the form of low‐dose‐rate treatment of skin malignancies or mycosis fungoides — through an off‐label use of the current beam in Developer mode using multileaf collimation. Such a use could render an orthovoltage treatment machine unnecessary for some treatments. Nanoparticles, especially gold‐based radiation therapies, could enhance the tumoricidal effects of treatment due to availability of low‐energy photon component in 2.5 MV spectrum.^(17)^

## V. CONCLUSIONS

In this paper we report our initial experiences with the 2.5 MV imaging beam available on the TrueBeam 2.0 linear accelerator. The beam output was stable over 5 months to within 1% and should be monitored monthly. The beam output was calibrated following the TG‐51 procedure. We recommend the beam symmetry, profile, and output be checked monthly, along with a monthly image quality constancy check. The beam possesses a lower average effective energy compared to a 6 MV treatment beam as used on conventional linear accelerators and demonstrates superior image quality for common imaging phantoms. A robust Winston‐Lutz test verifies that the 2.5 MV beam is coincident with the 6 MV treatment beam to within 0.2 mm. The measured $d_{\max}$ of approximately 5–6 mm, $\%\text{dd}{\left(10\right)}^{x}$ of 52% and $k_{Q}$ of unity were used for calibration with the TG‐51 protocol. The 2.5 MV imaging dose is controllable by the linac MU chambers, and offers the potential to optimize image quality and dosimetry from portal imaging applications.

## ACKNOWLEDGMENTS

A special thanks to Leo Moriarity, Allan Michaud, and Christian Bagg of the TBCC machine shop for their assistance in designing and constructing the film holder for the PDD measurements.

## COPYRIGHT

This work is licensed under a Creative Commons Attribution 3.0 Unported License.
